# Temporal differential effects of proinflammatory cytokines on osteoclastogenesis

**DOI:** 10.3892/ijmm.2013.1269

**Published:** 2013-02-05

**Authors:** SU-JIN MOON, INHYE E. AHN, HYERIN JUNG, HYOJU YI, JURYUN KIM, YOUNGKYUN KIM, SEUNG-KI KWOK, KYUNG-SU PARK, JUN-KI MIN, SUNG-HWAN PARK, HO-YOUN KIM, JI HYEON JU

**Affiliations:** 1 Division of Rheumatology, Department of Internal Medicine, School of Medicine, The Catholic University of Korea; 2 CiSTEM Laboratory, Convergent Research Consortium for Immunologic Diseases, Seoul St. Mary’s Hospital, The Catholic University of Korea, Seoul, Republic of Korea; 3 Department of Internal Medicine, The Methodist Hospital, Houston, TX, USA

**Keywords:** inflammation, osteoclast, receptor activator nuclear factor-κB ligand, interleukin-1β, interleukin-6, nuclear factor-κB

## Abstract

Bone destruction and inflammation are closely linked. Cytokines play an important role in inflammatory bone destruction by upregulating the receptor activator of nuclear factor-κB (NF-κB) ligand (RANKL). The direct role of cytokines that act in a non-RANKL-dependent manner has yet to be elucidated. The aim of this study was to investigate the direct osteoclastogenic properties of inflammatory cytokines at different time-points of osteoclastogenesis. Mouse bone marrow macrophages were stimulated with the macrophage colony-stimulating factor (M-CSF) and various concentrations of RANKL. Inflammatory cytokines, such as tumor necrosis factor (TNF)-α, interleukin (IL)-1β, IL-6, IL-17 and IL-23, were added to the culture system of osteoclastogenesis. Two time-points of cytokine treatment were set. The ‘early’ effect of each cytokine was investigated at the time of first RANKL treatment, whereas the ‘late’ effect was investigated 48 h after the first RANKL challenge. Osteoclast differentiation and function were assessed using an osteoclast marker [tartrate-resistant acid phosphatase (TRAP)] and by visualization of pit formation. A permissive level of RANKL was required for cytokine-associated osteoclastogenesis in all experiments. In the M-CSF/RANKL monocellular culture system, IL-1β enhanced and IL-6 decreased osteoclast formation in a dose-dependent manner, regardless of temporal differences. Other cytokines showed various responses according to the phase of osteoclast maturation and the concentration of each cytokine and RANKL. Furthermore, luciferase assays showed that both IL-1β and RANKL activated the NF-κB signaling pathway. Collectively, our data revealed that targeting IL-1β may be a promising strategy to inhibit inflammation-associated bone destruction and osteoporosis.

## Introduction

Progressive joint destruction, which is a pathognomonic characteristic of rheumatoid arthritis (RA), is caused by chronic inflammation and excessive osteoclastogenesis (1,2). Under physiological conditions, a balance between bone formation and resorption is maintained for skeletal homeostasis. In pathological states, such as RA, this balance is disrupted in favor of osteoclast-mediated bone resorption. Excessive osteoclastogenesis occurring at the frontline of the synovium-bone interface induces bone resorption (3). Metabolic activation of osteoclasts for potentiating the bone-resorbing ability requires ‘biochemical fueling’ from complex cellular interactions between cells of the osteoclast lineage and mesenchymal cells and lymphocytes (4–6).

These interactions are controlled by various cytokines and by the receptor activator of nuclear factor-κB (NF-κB) ligand (RANKL). A large number of cytokines have been shown to regulate osteoclast formation and function. In addition, a number of cytokines were recently shown to have a major influence on the ability of osteoclasts to resorb bone. RANKL is regarded as an essential cytokine that involves the interaction between the immune and bone systems. RANKL is necessary for the differentiation of osteoclast precursors into mature osteoclasts, and its deficiency is sufficient to block osteoclast formation completely (7,8). As a member of the tumor necrosis factor (TNF) superfamily, RANKL is expressed mainly by osteoblasts. In pathological conditions, including RA, this role is deferred to active fibroblast-like synoviocytes and T cells residing in the inflamed joint (9,10). RANKL expression is upregulated in, and constitutes an important prerequisite for, osteoclast differentiation in animal models of RA and in rheumatoid synovial tissue (11,12). In addition, RANKL expression is upregulated by inflammatory cytokines, such as TNF-α, interleukin-1β (IL-1β), IL-6, IL-17 and IL-23 (13–17).

Cytokines enhance osteoclastogenesis indirectly by upregulating RANKL expression. However, the theories on RANKL-dependent osteoclastogenesis are controversial. Certain authors demonstrated that TNF-α, IL-1β and IL-6 induced osteoclast differentiation directly, in a RANKL-independent manner (18–20), whereas others showed that a permissive level of RANKL is necessary for TNF-α-induced osteoclastogenesis, thus concluding that TNF-α alone cannot induce osteoclast formation (21).

We considered that this controversy regarding cytokine-induced, RANKL-independent osteoclastogenesis stems from the fact that these previous experiments were performed under different conditions. To minimize the confounding factors that may arise from the experimental system of osteoclastogenesis, we adopted a monocellular culture system instead of a coculture system, consisting of osteoblasts and bone marrow cells. Mouse bone marrow-derived macrophages (BMMs) were stimulated with the macrophage colony-stimulating factor (M-CSF) and various concentrations of RANKL. Inflammatory cytokines (TNF-α, IL-1β, IL-6, IL-17 and IL-23) were added to the osteoclastogenesis culture system at two different time-points: at 0 h after the stimulation with RANKL (early phase), when undifferentiated osteoclast precursors were predominant, and at 48 h after the stimulation with RANKL (delayed phase), when half-matured osteoclasts appeared. In the present study, we identified the direct influence of each inflammatory cytokine on RANKL-induced osteoclast differentiation and its effect on the bone-absorbing ability of osteoclasts using a monocellular culture system.

## Materials and methods

### Reagents and proteins

Recombinant mouse IL-1β, IL-6, IL-17, IL-23 and TNF-α were purchased from R&D Systems (Minneapolis, MN, USA). Soluble RANKL was purchased from PeproTech (London, UK). The tartrate-resistant acid phosphatase (TRAP) kit was purchased from Sigma-Aldrich (St. Louis, MO, USA) and a Cell Counting kit (CCK)-8 was purchased from Dojindo Laboratories (Kumamoto, Japan). The pNF-κB-Luc, pAP-1-Luc, CREB-Luc and pNFAT-Luc plasmids were purchased from Stratagene (La Jolla, CA, USA).

### Animals

Six-week-old male DBA/1J mice were purchased from SLC, Inc., (Shizuoka, Japan). Animals were maintained under specific pathogen-free conditions at the Institute of Medical Science, the Catholic University of Korea, and were fed standard mouse chow (Ralston Purina) and water *ad libitum*. All experimental procedures were examined and approved by the Animal Research Ethics Committee of the Catholic University of Korea, which conforms to all USA National Institutes of Health guidelines (approval ID 2011-0062-02).

### In vitro osteoclastogenesis

**Figure 1:**
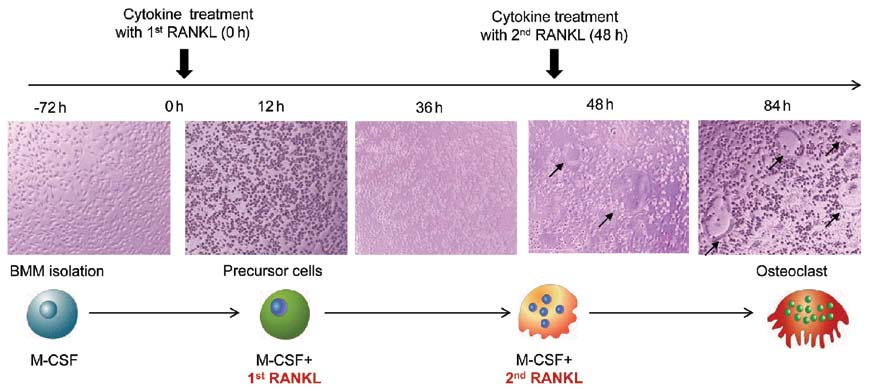
Schematic description of the osteoclastogenesis system and cytokine treatment. Early and late challenges with cytokines were performed simultaneously with the first RANKL stimulation (at 0 h) and the second RANKL stimulation (after 48 h).

BMMs were isolated from the tibia and femur of 6-week-old DBA/1J male mice by flushing the bone marrow cavity with minimum essential medium-α (α-MEM; Invitrogen, Carlsbad, CA, USA). The cells were then centrifuged, exposed to hypotonic ACK buffer (0.15 mM NH_4_Cl, 1 mM KCO_3_ and 0.1 mM EDTA, pH 7.4) at room temperature for 30 sec to remove red blood cells, and incubated with α-MEM containing penicillin/streptomycin and 10% heat-inactivated fetal bovine serum for 12 h to separate the floating and adherent cells. Floating cells were collected, suspended in α-MEM, counted, seeded on 24-well plates (Nalge Nunc International, Naperville, IL, USA) at 2×10^5^ cells/well, and cultured in α-MEM in the presence of 30 ng/ml M-CSF for 3 days to form macrophage-like osteoclast precursor cells. After 3 days, adherent cells were used as BMMs after washing out the nonadherent cells, including lymphocytes. These osteoclast precursor cells were further cultured in the presence of 30 ng/ml M-CSF, various concentrations of RANKL and cytokines to generate osteoclasts. The RANKL concentrations used in the present study ranged from 1 to 100 ng/ml. Various concentrations of cytokines and RANKL were added into the osteoclastogenesis culture system at two different time-points; early cytokine treatment was performed with the first RANKL administration, and late cytokine treatment was performed on Day 2, when the second RANKL treatment was administered to the osteoclast culture media. On Day 2, the media were replaced with fresh medium containing M-CSF, RANKL, and cytokines. Precursor cells began to fuse between 36 and 48 h and mature osteoclasts were observed at 60 h after RANKL stimulation (Fig. 1).

### TRAP staining

A commercial kit (catalog no. 387A; Sigma-Aldrich) was used according to the manufacturer’s instructions, omitting the counterstaining with hematoxylin. TRAP-positive cells containing three or more nuclei were counted as osteoclasts. TRAP-positive cells were counted three times without knowledge of the previous counts of osteoclasts.

### Bone resorption assay

To assess the effect of each cytokine on RANKL-induced bone resorption, an osteoclast bone resorption assay was performed using a commercially available bone resorption assay kit (Cosmo Bio Co., Ltd., Tokyo, Japan). BMMs were cultured on CaP-coated plates in the presence of M-CSF (30 ng/ml) and RANKL (50 and 100 ng/ml) in the presence or absence of various concentrations of cytokines for 7 days. Cytokine treatment was performed at different time-points: at 0 h from the first RANKL treatment and at 48 h after the first RANKL treatment, in accordance with previous TRAP staining experiments. The cells were removed from the plates (by wiping their surface), followed by staining of the slides with toluidine blue (1 μg/ml). The resorbed areas on the plate were visualized using light microscopy.

### Cell proliferation assays

Cytotoxicity assays were conducted using a CCK-8 (Dojindo Laboratories, Kumamoto, Japan), which produces a highly water-soluble formazan dye. Cells were seeded in 96-well plates (5×10^4^ cells/well, 100 μl/well) and cultured in the presence of M-CSF, RANKL, and the indicated concentrations of various cytokines. At the end of the culture period, 10 μl of the CCK-8 reagent was added to each well. After 1 h of incubation at 37°C, the absorbance was determined at 450 nm using a microplate reader (Vmax; Molecular Devices, Palo Alto, CA, USA).

### Transfection and luciferase reporter gene activity assay

For transfection, RAW264.7 cells were seeded in 6-well plates (1×10^6^ cells/well) and transfection with pNF-κB-Luc, pAP-1-Luc, CREB-Luc and pNFAT-Luc plasmids was performed using FuGene^®^ HD according to the manufacturer’s instructions (Roche Diagnostics, Mannheim, Germany). After 48 h, the medium was replaced with fresh medium and cells were stimulated with various cytokines. The cells were incubated for an additional 24 h and luciferase activity was determined using a luciferase assay kit (Promega, Madison, WI, USA). Luciferase activities were normalized to the corresponding β-galactosidase levels (Promega).

### Statistical analysis

Data were analyzed using the SPSS software version 16.0 (SPSS Inc., Chicago, IL, USA). Data are presented as the means ± standard deviation (SD). Results were analyzed using the Kruskal-Wallis test, followed by the Mann-Whitney U-test. P-values <0.05 were considered to indicate statistically significant differences.

## Results

### IL-1β induces osteoclastogenesis in BMMs in a dose-dependent manner

**Figure 2:**
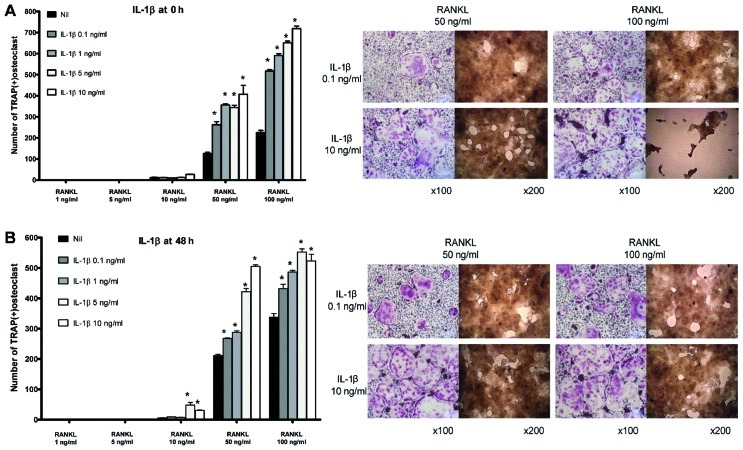
Osteoclastogenic effect of IL-1β on osteoclast formation and their bone-absorbing ability. BMMs were treated with various doses of RANKL. TRAP-positive multinucleated cells with three or more nuclei were counted as osteoclasts. In both the ‘early’ (A) and the ‘late’ (B) phase of osteoclastogenesis, IL-1β increased the number of osteoclasts and their bone-absorbing ability in a dose-dependent manner. Values are the means ± SD of 3 independent experiments. ^*^P<0.001 compared with Nil. Nil, control group.

Osteoclastogenesis was not induced either in the absence of RANKL or in the presence of RANKL at a concentration <50 ng/ml. Thus, a permissive level of RANKL ( ≥50 ng/ml) was required for osteoclastogenesis. Both in the ‘early’ (Fig. 2A) and ‘late’ phases (Fig. 2B) of osteoclastogenesis, IL-1β increased the number of osteoclasts and their bone-absorbing ability. At all given concentrations of IL-1β, the number of TRAP-positive multinucleated cells was increased in its presence compared with the control group. In addition, the pro-osteoclastogenic effect of IL-1β appeared to be dose-dependent in both phases of osteoclastogenesis.

### IL-6 decreases osteoclastogenesis in BMMs in a dose-dependent manner

**Figure 3:**
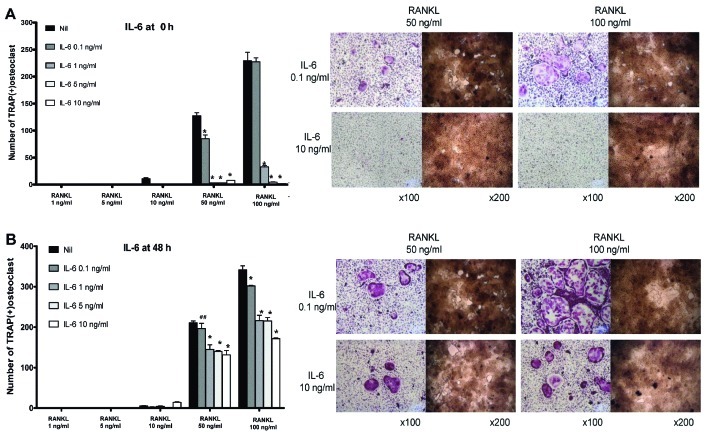
Suppression of osteoclastogenesis by IL-6. (A) In the ‘early’ phase of osteoclastogenesis, osteoclastogenesis is almost entirely suppressed by IL-6 administration from the concentration of 1 ng/ml. (B) In the ‘late’ phase of osteoclastogenesis, IL-6 also decreased the number of osteoclasts and their bone-absorbing ability in a dose-dependent manner. Values are the means ±SD of 3 independent experiments. ^##^P<0.01 and ^*^P<0.001 compared with Nil. Nil, control group.

In contrast to IL-1β, IL-6 decreased osteoclast formation and bone-absorption ability, both in the ‘early’ and ‘late’ phases of osteoclastogenesis. In the early phase of osteoclastogenesis, IL-6 decreased the number of multinucleated osteoclasts markedly from a dose of 1 ng/ml (Fig. 3A). In addition, in the late phase of osteoclastogenesis, IL-6 reduced the number of TRAP-positive multinucleated cells at all the given concentrations of IL-6 in a dose-dependent manner (from 0.1 to 10 ng/ml) (Fig. 3B).

### Suppressive effects of IL-17 on the late phase of osteoclastogenesis

**Figure 4:**
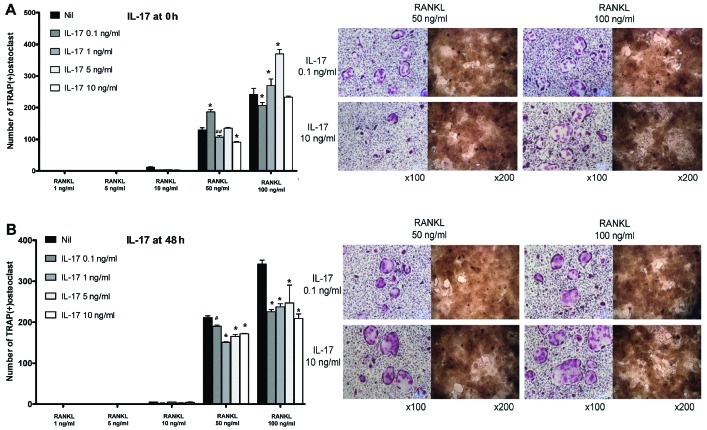
Suppressive effects of IL-17 on the late phase of osteoclastogenesis. (A) IL-17 exhibited different effects on osteoclastogenesis in the ‘early’ phase of osteoclastogenesis at the given concentrations. (B) IL-17 yielded suppressive effects on osteoclastogenesis at all concentrations (from 0.1 to 10 ng/ml) in the ‘late’ phase of osteoclastogenesis. Values are the means ± SD of 3 independent experiments. ^#^P<0.05 and ^*^P<0.001 compared with Nil. Nil, control group.

In BMM-induced osteoclastogenesis, IL-17 acted differently on the time to challenge. In the early phase, IL-17 did not yield coherent results (Fig. 4A). In certain conditions, IL-17 appeared to act as an anti-osteoclastogenic factor, while in other conditions, IL-17 increased osteoclastogenesis. Conversely, challenge with IL-17 in the late phase of osteoclastogenesis decreased the number of osteoclasts at all given concentrations (from 0.1 to 10 ng/ml), although the pattern was not dose-dependent (Fig. 4B).

### Contrary effect of IL-23 according to the phase of osteoclastogenesis

**Figure 5:**
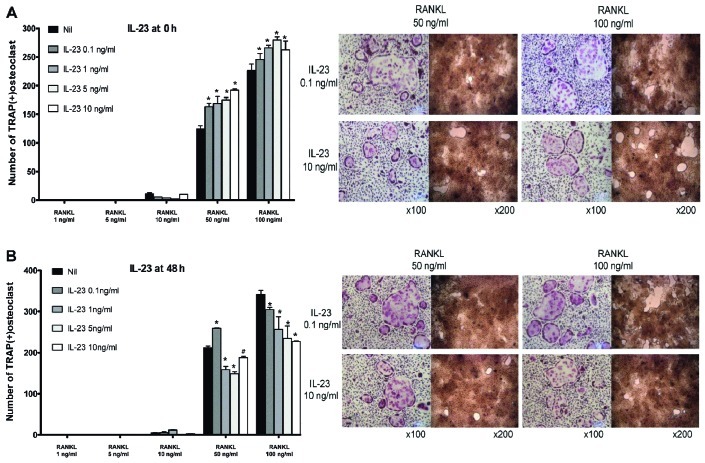
Contrary effects of IL-23 according to the time-points of cytokine administration. (A) In the ‘early’ phase of osteoclastogenesis, IL-23 enhanced osteoclastogenesis in a dose-dependent manner. (B) However, in the ‘late’ phase of osteoclastogenesis, IL-23 (except at the concentration of 0.1 ng/ml) suppressed osteoclastogenesis. Values are the means ± SD of 3 independent experiments. ^#^P<0.05 and ^*^P<0.001 compared with Nil. Nil, control group.

In the early phase of osteoclastogenesis, IL-23 enhanced osteoclast formation and its bone-absorption ability in a dose-dependent manner (Fig. 5A). In the late phase, however, IL-23 suppressed osteoclast formation. IL-23 exhibited a pattern that was similar to that of IL-1β when it was applied in the late phase of osteoclastogenesis (Fig. 5B), although the osteoclastogenic potency of IL-23 was significantly lower than that of IL-1β.

### Pro-osteoclastogenic effects of TNF-α in the early phase of osteoclastogenesis

**Figure 6:**
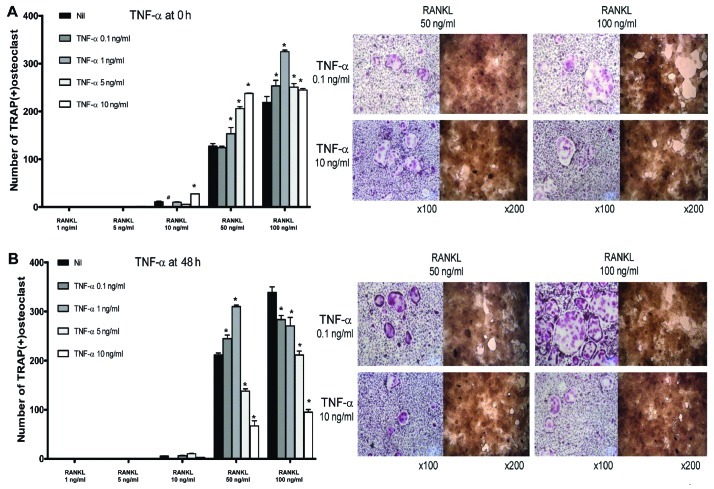
Osteoclastogenic effect of TNF-α. (A) In the ‘early’ phase of osteoclastogenesis, TNF-α enhanced osteoclastogenesis, although the effect was not dose-dependent. (B) In the ‘late’ phase of osteoclastogenesis, osteoclasts were less differentiated by an administration of TNF-α in a dose-dependent manner (when BMMs were stimulated with 100 ng/ml of RANKL). Values are the means ± SD of 3 independent experiments. ^*^P<0.001 compared with Nil. Nil, control group.

Osteoclast precursor cells exhibited complex responses to treatment with TNF-α. Stimulation of BMMs with a lower concentration of RANKL (50 ng/ml) in the early phase of osteoclastogenesis resulted in enhanced osteoclastogenesis following the challenge with TNF-α, in a dose-dependent manner. At a higher concentration of RANKL (100 ng/ml), TNF-α enhanced osteoclast formation, although dose-dependency was not observed (Fig. 6A). In the late phase, TNF-α seemed to act as an anti-osteoclastogenic factor at a higher concentration of RANKL (100 ng/ml). At a lower concentration of RANKL (50 ng/ml), BMMs responded to TNF-α in a different way. At lower concentrations of TNF-α (0.1 and 1 ng/ml), osteoclast formation was increased. However, the number of osteoclasts was decreased at higher doses of TNF-α (5 and 10 ng/ml) (Fig. 6B).

### IL-1β and RANKL activate NF-κB at an early phase, with a similar pattern

**Figure 7:**
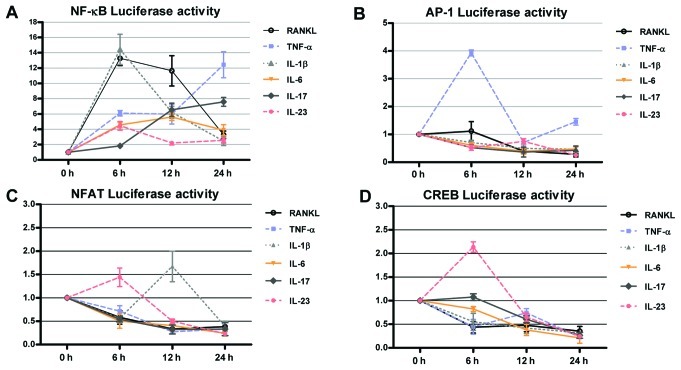
Luciferase activity of transcriptional factors during cytokine-stimulated osteoclastogenesis. (A) All the cytokines induced enhanced NF-κB activity, although the patterns were different. RANKL, IL-1β and IL-23 induced peak level of luciferase activity of NF-κB at 6 h after administration of these cytokines. The peak level of NF-κB luciferase activity was shown at 12 h after IL-6 administration. In TNF-α and IL-17, NF-κB activity reached peak levels at 24 h. (B) The luciferase activity of AP-1 was increased only at 6 h after TNF-α administration. (C) The luciferase activity of NFAT showed a tendency to be decreased by the stimulation with cytokines, except IL-23 at 6 h and IL-1β at 12 h. (D) The luciferase activity of CREB was increased only at 6 h after the IL-23 challenge. Other cytokines appeared to reduce CREB activity.

To assess the changes in NF-κB, AP-1, NFAT and CREB activity following treatment with the cytokines mentioned above, transient transfection was performed using luciferase constructs. RAW264.7 cells were incubated in the presence of RANKL, TNF-α, IL-1β, IL-6, IL-17 and IL-23. A luciferase assay was performed serially from 6 to 24 h after stimulation with each cytokine. IL-1β yielded an early increase of NF-κB, mirroring the RANKL-induced signaling pathway (Fig. 7A). Treatment with other cytokines also increased NF-κB activity, although the degree of this increase and the peak time-points varied. Other cytokines exhibited specific patterns of activation of the signaling pathway (Fig. 7B-D).

### Treatment with the cytokines does not have cytotoxic effects

**Figure 8:**
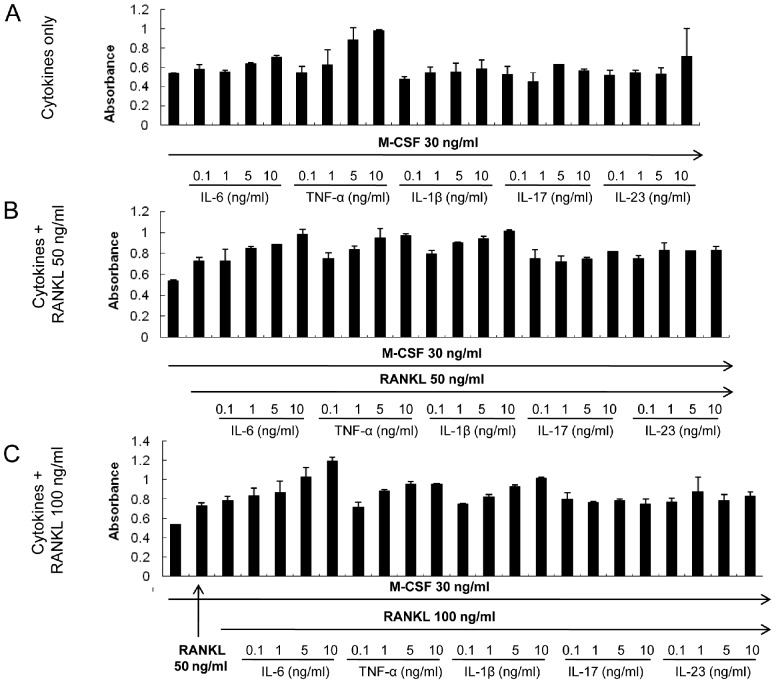
Cell viability analysis by CCK-8 kit. CCK-8 assay showed that various cytokines (A), cytokines and RANKL 50 ng/ml (B), cytokines and RANKL 100 ng/ml (C) did not cause apoptosis.

A suppressive effect on osteoclastogenesis was observed for some cytokines: IL-6, IL-17, IL-23 and TNF-α. CCK-8 assay was performed to ensure that the inhibitory effect of those cytokines was not due to enhanced apoptosis or cytotoxicity. None of the cytokines resulted in apoptotic or toxic effects in BMMs at the concentrations used in the present experiments (Fig. 8). Moreover, IL-6 stimulated the proliferation of BMMs in a dose-dependent manner, a tendency that was augmented with increasing RANKL concentrations (Fig. 8B and C).

## Discussion

In the present study, we investigated the effect of several cytokines on osteoclastogenesis using a monocellular culture system, and clarified whether the proinflammatory cytokines IL-1β, IL-6, IL-17, IL-23 and TNF-α have a similar effect on osteoclastogenesis, as they are known to be similar types of proinflammatory cytokines. However, our findings demonstrated that IL-1β increased the number of osteoclasts and their bone-absorption ability, whereas IL-6 yielded the opposite effect.

One of the challenges of comparing certain characteristics of a molecule with accuracy, is that a strict control of confounding factors is required. Even if the features of the molecules are successfully compared, applying this approach to an *in vivo* system and in physiological conditions is difficult. To minimize the confounding factors and to determine the pure effect of cytokines on osteoclastogenesis, we adopted a monocellular culture system using only osteoclast precursor cells derived from mouse bone marrow. A coculture system is composed of osteoblasts and bone marrow cells stimulated with vitamin D3 and prostaglandin E2 (22). The existence of various cell components other than precursor cells produces several confusing variables that do not reflect the pure effect of cytokines.

The cytokine milieu determines the effects of inflammation on bone. There is extensive research on the role of proinflammatory cytokines in bone homeostasis (23–25). Blockade of the inflammatory cytokines results in the control of chronic inflammation as well as in protective effects on bone. In general, TNF-α, IL-1β and IL-6 promote bone resorption via either the direct or indirect promotion of osteoclastogenesis. TNF-α inhibition has been successful in the therapy of patients with RA, yielding control of inflammation as well as retardation of structural changes in the joints involved (26). Moreover, suppression of IL-1 and IL-6 resulted in the marked suppression of inflammation and joint destruction in patients with RA (27–29). The IL-23/IL-17 axis was previously demonstrated to be a potential linker between inflammation and bone loss. Inhibition of IL-23 and IL-17 attenuated bone erosion in an animal model of arthritis (30,31). Based on the beneficial effects of anticytokine treatments observed in human and animal trials, it could be assumed that the above-mentioned cytokines have osteoclastogenic properties. However, the role of proinflammatory cytokines on osteoclast precursor cells remains unclear.

Therefore, we determined whether the various cytokines have different effects on osteoclast precursor cells in the context of their maturation stages. Two different time-points were selected for cytokine stimulation in osteoclast precursor cells; the first was set at an early time after RANKL stimulation, when precursor cells had not yet initiated the differentiation process, and the other was set at a later time (48 h after the initial stimulation with RANKL), when osteoclast precursor cells had initiated the differentiation process, i.e., ‘committed osteoclasts’. IL-1β and IL-6 had a consistent effect on precursor cells, regardless of maturation status. IL-23 and TNF-α yielded different responses depending on maturation status. IL-23 and TNF-α may be helpful for osteoclastogenesis when the fate of precursor cells is not committed.

We postulated that a decreasing number of mouse BMMs may reflect the suppressive effect of some cytokines, such as IL-6 and TNF-α, due to cytotoxic effects of cytokines and apoptosis. A CCK-8 assay demonstrated that cytokines did not induce apoptosis or cellular toxicity at the given concentrations. The suppressive tendency of some cytokines, such as IL-17, IL-23 and TNF-α, may have also resulted from a disruption in the balance of BMMs, with a shift towards activated macrophages. If a larger proportion of BMMs is differentiated into activated macrophages in response to cytokine stimulation, a smaller proportion of candidate cells can fuse and form mature osteoclasts, leading to a decreased number of osteoclasts.

The mechanism underlying the pro-osteoclastogenic potential of IL-1β could be partly explained by the sharing of the early NF-κB signaling pathway with RANKL. RANKL and IL-1β led to the early upregulation of NF-κB in RAW264.7 cells. NFAT is a candidate molecule that could be activated by RANKL stimulation. However, stimulation by RANKL did not lead to the activation of the signaling NFAT. Therefore, we analyzed NFAT signaling again by dissecting it into NFATc1 and NFATc2. Notably, NFATc1 and NFATc2 exhibited reciprocally activating patterns (data not shown). At the early time-point of 6 h, the NFATc1/NTATc2 ratio increased following RANKL stimulation. At the later time-point of 24 h, the ratio was reversed. RANKL also decreased the temporal ratio of NFATc1 (24/6 h) and increased the ratio of NFATc2 (24/6 h) in a dose-dependent manner. These results indicate that NFATc1 plays an important role during early RANKL-induced osteoclastogenesis.

In the present study, we demonstrated that cytokines have specific characteristic osteoclastogenic properties. Cytokines exhibited their osteoclastogenesis-related activity only when a permissive level of RANKL existed. IL-1β enhanced osteoclastogenesis, regardless of RANKL concentration or the maturation status of precursor cells. IL-6 interrupted the process of osteoclastogenesis in osteoclast precursor cells under all the given conditions. IL-17 slightly favored a suppressive effect in the late stage of osteoclastogenesis. IL-23 and TNF-α appeared to have osteoclastogenic properties in the early stage of osteoclastogenesis, when BMMs are ‘just’ osteoclast precursor cells.

In conclusion, IL-1β was the most potent osteoclastogenic cytokine among the proinflammatory molecules studied, and IL-6 disturbed osteoclast formation, as well as the bone-absorption ability of these cells. We consider this a notable phenomenon, as shown in *in vitro* experiments. Nevertheless, our *in vitro* results should not be extended for more complex *in vivo* biological systems.
